# The Epidemic of Obesity in a Pandemic Era: Urgency to Invest in Adolescent Health

**DOI:** 10.3390/children8070608

**Published:** 2021-07-19

**Authors:** Flora Bacopoulou

**Affiliations:** University Research Institute of Maternal and Child Health & Precision Medicine, and UNESCO Chair on Adolescent Health Care, National and Kapodistrian University of Athens, Aghia Sophia Children’s Hospital, 8 Levadias Street, 11527 Athens, Greece; bacopouf@hotmail.com; Tel.: +30-210-7467476

In September 2015, United Nations’ 193 member states signed up to the Sustainable Development Goals (SDGs) of the global development agenda 2030. Given the explosion in the prevalence of obesity throughout the world, SDG 3.4 aims to reduce premature mortality from non-communicable diseases (NCDs) with a focus on obesity-related pathologies such as diabetes mellitus type 2 and cardiovascular disease. 

Many countries are falling behind on their commitment not only to reduce premature morbidity and mortality from NCDs but also to place adolescents in the center stage of the health development agenda. Children and adolescents with overweight or obesity are expected to have even higher body mass index (BMI) as adults [1]. Also, adolescent obesity is strongly related to ‘adult’ metabolic syndrome pathologies such as insulin resistance, hypertension, dyslipidemia, non-alcoholic fatty liver disease, diabetes mellitus type 2, thus increasing the risk of NCDs in adulthood. 

The purpose of this special issue was to summarize new information from experts in the field of obesity in children and mainly in adolescents. 

Over the last decade, extensive research on the pathogenesis of obesity supports the role of complex biomolecules that mediate the communication between adipose tissue and other cells/organs. Maligianni et al. [2] reviewed the latest data from human clinical studies and animal models on the genetic and epigenetic contributors to obesity. This review showcased the emerging era of exosomal endocrinology [3] in the pathophysiology of obesity and described the functions of the novel adipose-derived exosomes and their non-coding RNAs, as well as their potential role in childhood and adolescent obesity.

Apart from evolutionary and developmental factors, dietary, physical, psychosocial, and other environmental factors, can all impact negatively energy homeostasis and trigger obesity in children and adolescents. 

Overlooked psychosocial factors such as the area of residence, anxiety, melancholic depression, smoking, school performance, and maternal occupation were highlighted by Andrie et al. [4] as contributors to overweight/obesity in 414 adolescents in Greece, a country with a high prevalence of adolescent obesity. 

Despite young people’s alarming rates of overweight/obesity along with online activities worldwide, few data are published on the associations between cyberbullying and overweight/obesity. In a cross-sectional study of 8785 adolescents from randomly selected schools across seven European countries, Sergentanis et al. [5] revealed country-specific associations between cyberbullying victimization and overweight/obesity. The association of cyberbullying victimization with overweight was prominent in Germany, whereas an association with obesity emerged only in Iceland. Interestingly, no such associations were found for adolescents from Greece, Spain, Romania, Poland, or the Netherlands.

Obesity, especially that starting from an early age, increases the risk of reproductive ‘adult’ pathologies as well. Giannouli et al. [6] presented the third case of vulvar venous varicosities in an adolescent girl with morbid obesity.

Finally, obesity has evolved as a major risk factor for severe coronavirus disease 2019 (COVID-19) in all age groups. The review by Stavridou et al. [7] underlined the mutual relation of COVID-19 and obesity in children, adolescents, and young adults due to the inevitable restrictions imposed by the pandemic. 

The COVID-19 pandemic has reminded us of the value of health promotion and revealed the failure of the exclusive disease-based model of medicine. This is an opportunity in history to increase our efforts for the transition from disease-centered to lifestyle-directed medicine. There is an urgent need to realize that adolescents and young people are our best chance to boost action against the epidemic of NCDs for a sustainable world, as adolescents of today will be the policymakers of 2030 that will determine future population health. 

## Data Availability

Not applicable.
